# Shark IgNAR: The Next Broad Application Antibody in Clinical Diagnoses and Tumor Therapies?

**DOI:** 10.3390/md21090496

**Published:** 2023-09-16

**Authors:** Xiaofeng Jiang, Ling Sun, Chengwu Hu, Feijian Zheng, Zhengbing Lyu, Jianzhong Shao

**Affiliations:** 1 College of Life Sciences, Zhejiang Sci-Tech University, Hangzhou 310018, China; 202120801057@mails.zstu.edu.cn (L.S.); 202220901018@mails.zstu.edu.cn (C.H.); zhengbingl@zstu.edu.cn (Z.L.); 2 Zhejiang Provincial Key Laboratory of Silkworm Bioreactor and Biomedicine, Zhejiang Sci-Tech University, Hangzhou 310018, China; 3 Jiangsu Baiying Biotech Co., Ltd., Taizhou 225300, China; zhengfeijian@biointron.com; 4 College of Life Sciences, Zhejiang University, Hangzhou 310058, China

**Keywords:** single-domain antibody, immunodiagnostic, shark, VNAR, antibody–drug conjugate, anti-tumor therapy

## Abstract

Antibodies represent a relatively mature detection means and serve as therapeutic drug carriers in the clinical diagnosis and treatment of cancer—among which monoclonal antibodies (mAbs) currently occupy a dominant position. However, the emergence and development of small-molecule monodomain antibodies are inevitable due to the many limitations of mAbs, such as their large size, complex structure, and sensitivity to extreme temperature, and tumor microenvironments. Thus, since first discovered in Chondroid fish in 1995, IgNAR has become an alternative therapeutic strategy through which to replace monoclonal antibodies, thus entailing that this novel type of immunoglobulin has received wide attention with respect to clinical diagnoses and tumor therapies. The variable new antigen receptor (VNAR) of IgNAR provides an advantage for the development of new antitumor drugs due to its small size, high stability, high affinity, as well as other structural and functional characteristics. In that respect, a better understanding of the unique characteristics and therapeutic potential of IgNAR/VNAR in clinical and anti-tumor treatment is needed. This article reviews the advantages of its unique biochemical conditions and molecular structure for clinical diagnoses and novel anti-tumor drugs. At the same time, the main advantages of the existing conjugated drugs, which are based on single-domain antibodies, are introduced here, thereby providing new ideas and methods for the development of clinical diagnoses and anti-tumor therapies in the future.

## 1. Preface

Cancer and chronic inflammatory diseases are currently the main indications for antibody therapy; this is due, in part, to the systemic accessibility of the target antigens. To date, the clinically approved therapeutic drug market is still dominated by mAbs of the traditional IgG type, and they continue to show great commercial impact and sustainable market growth [1]. The intrinsic advantages of mAbs include high levels of specificity in terms of a target, reduced off-site toxicity, the efficacy benefits of induced cell death, and a long serum half-life [2,3]. Despite this success, biological limitations and economic barriers remain obstacles to overcome in the many forms of mAb drugs used in clinical diagnoses and tumor therapies [2,4].

Antibody fragments with a smaller molecular weight, which have been shown to have a more uniform distribution than mAbs in tissues, can improve diffusivity and vascular permeability [5]. In diagnostic and therapeutic applications, smaller and more flexible therapeutic antibody formats with exciting therapeutic potential have been considered and developed for optical imaging both in vitro and in vivo [6], viruses [7,8], tumors [9], and other human disease therapies [10].

In 1989, two variable domains of an antibody heavy chain with antigen-binding abilities were screened out from mice by Ward et al. [11]; they were only able to determine the heavy chains and named them single-domain antibodies (sdAbs). An antibody family called the heavy-chain antibodies (HCAbs), which naturally lack light chains, was initially found in the humoral immune system of mammalian camelids in 1993 [12]. Two years later, an immunoglobulin (Ig)-based new antigen receptor (IgNAR) of the same type was also found in cartilaginous fish in 1995 [13]. As a new therapeutic strategy to replace monoclonal antibodies, the major types of naturally occurring single-domain antibodies are, thus far, derived from the antigen-binding variable domain of camelids and sharks, which are referred to as the variable domain of the heavy chain of heavy-chain antibodies (VHHs) and variable new antigen receptors (VNARs), respectively. These both function as two members of the most ancient antigen recognition receptors [14,15,16]. 

**Figure 1:**
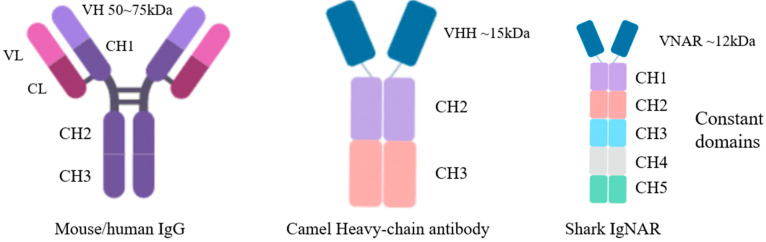
Size and structure of IgG and Ig–like biologics. Schematic representation of molecular models depicting the relative structure and size of different antibodies showing different options for treatment.

Structurally, the conventional antibodies (IgG) are mostly heterodimers of 150–160 kDa, with two heavy chains (VHs) and two light chains (VLs) [17]. By contrast, the form of the IgNAR molecule is a heavy-chain dimer, and each heavy chain of IgNAR comprises five constant domains that follow a single variable domain, VNAR. The VNARs of IgNAR are truly smaller, with a molecular mass of ~12 kDa, than the camel-derived monodomain antibody because each carries only two CDRs (CDR1 and CDR3) and a deleted CDR2 region (Figure 1). In addition, VNARs have two additional high-variable loops (hypervariable 2 and hypervariable 4), and their small and simple domain generates four diversity loops [14,18]. Although CDR1 and CDR3 are considered the two major determinants for antigen binding by VNAR domains, other hypervariable regions—such as HV2 and HV4—show an increased frequency of somatic mutations, indicating their potential involvement in antigen recognition [19]. Due to their unique structures and binding modes, shark VNARs may have unique therapeutic potential. 

## 2. Structural Characteristics and Advantages of IgNAR

### 2.1. Limitations of mAbs 

Certain limitations in monoclonal antibody treatments have existed in the clinical diagnosis and treatment stages. First, mammalian IgG, as a drug, is unstable and orally unachievable; at the same time, it is often blocked when penetrating solid tumors [20] and crossing the blood–brain barrier (BBB) [21]. Second, due to the size of mAbs, which are approximately 150 kDa, the action site within the human body is limited to the cell surface molecules with minimal tissue penetration, as well as a variety of antigens, including epitopes located in clefts on protein surfaces (e.g., enzyme active sites) that are non-accessible [22,23], thus resulting in poor binding affinity (approximately ten times weaker than VNAR) [19] and low tissue infiltration ability [24]. Finally, the inconvenience, discomfort, and cost of treatment that mAb drug application brings considerably affect the patients. With traditional mAb drug treatments, inconsistent clinical effects exist in different diseases and patients. Moreover, it is ineffective for medium- and advanced-stage cancer patients. In addition, the high cost of treatment is required for producing complex, large globular glycoproteins [2]. In addition, due to the sensitivity of mAbs to temperature and humidity, maintaining their optimal performance is difficult, particularly when the drugs must be used in developing resource-poor countries with insufficient electricity supply [4,25]. 

In addition to its own limitations, a potential predominance of shark-derived VNAR therapeutic efficacy and response over that of mAbs has been observed in emerging viruses or new acute infectious diseases [7,26]. The COVID-19 pandemic, caused by severe acute respiratory syndrome coronavirus 2 (SARS-CoV-2), has led to a devastating global health crisis. SARS-CoV-2 has rapidly evolved into a highly infectious variant around the world. Researchers have suggested that the novel variants may reduce the sensitivity of SARS-CoV-2 to antibody therapies and vaccines, which would then complicate the development of antibodies [27]. Finding facilities equipped to administer an IV infusion that can manage anaphylaxis while not exposing uninfected patients to SARS-CoV-2 may be difficult [28]. 

Therefore, due to the limitations of traditional antibodies in clinical applications, developing diagnostic reagents and therapeutic drugs based on new types of antibody molecules is necessary.

### 2.2. Advantages of Shark-Derived VNAR

Newly developed biologics should overcome the inherent limitations of traditional antibodies. Furthermore, they have the ability to quickly cross the tissue barrier while retaining the specificity and affinity of antibodies, making them a meaningful supply for current monoclonal antibody therapies. Meanwhile, the activity of antibody drugs in vivo should be further optimized to increase their efficacy and expand their clinical applications. One of the candidate biological inhibitors with potential is based on the discovery of VNAR, which was found in the antigen-binding variable region of IgNAR in chondroid fish (Table 1). It has multiple natural and downstream attributes, such as small size, high solubility, thermal stability, folding ability, good tissue permeability in vivo, reduced propensity to aggregate, and high target-binding ability. VNARs have become attractive candidates for drug development due to these attributes.

#### 2.2.1. Structural Characteristics of Shark Monodomain Antibodies

The immunoglobulin neoantigen receptor antibody (IgNAR) from sharks is a heavy-chain protein dimer without associated light chains [29]. The specificity of the molecules is defined by the variable region of IgNAR, and VNAR types are defined in terms of cysteine number, CDR3 length, and amino acid variability. The single-domain natural lack of CDR2 in VNARs heightens the requirement for CDR1 and CDR3 to provide specific and high-affinity binding to prospective antigens [30]. It was observed that the VNAR domain with four antigen-binding coils over a single chain possessed the ability to bind antigens with relatively higher affinity than conventional antibodies containing six loops across two chains [31,32]. 

**Figure 2:**
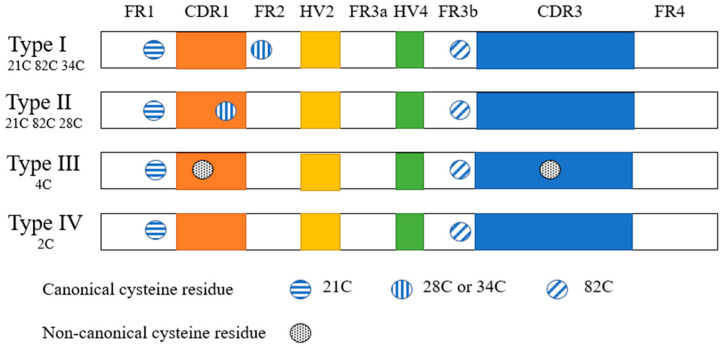
Illustration of VNAR isotypes showing the similarities and differences between different isotypes of VNAR domain. This schematic is based on the specific sequence and classification methods by Feng et al. [15].

In a natural VNAR phage library constructed from six naive nurse sharks [15], the presence of two canonical cysteines located at amino acids 21 and 82 was used as a key criterion for type I-IV VNAR; moreover, the sequences that did not contain one or both cysteines were considered other types (Figure 2). The high variability in CDR3 length and cysteine amount is critical for VNAR diversity because binding diversity is mainly dependent on the CDR3 structures [15]. Different from conventional antibodies, VNAR has a CDR2 region that is replaced by short-chain HV2 [29]. Therefore, to compensate for this condition, sequence diversity is reflected in the CDR3 region. VNARs encode unusually long and structurally complex CDR3s, which display a high degree of variability, to compensate for the reduced size of variable regions in IgNARs [33]. CDR3, which is more variable in terms of sequence, length, and conformation, plays a key role in antigen recognition. It has a maximum of 100+ amino acid residues [34], an unusual topological structure (which can bind relatively hidden epitopes of target antigens, such as pockets or grooves), and the ability to penetrate rapidly [35]. Each VNAR covers less area than standard bivalent antibodies but still maintains precise target specificity. Ubah et al. proved that two amino acid residues from the HV2 region are involved in the association of VNAR with SARS-CoV-2 receptor-binding domains (RBDs) in addition to the binding of the CDR region with the RBD. Their study provided additional evidence for the functional significance of the HV2 domain [27]. 

#### 2.2.2. Biochemical Characteristics of IgNAR 

VNARs have high chemical and thermal stability. Influenced by the critical evolutionary environment, IgNARs are resistant to chemical denaturants that can be more stable than classical antibodies. This option is attractive for drug delivery, diagnostics, and imaging. It is stable in the blood of sharks that contain 350 mmol/L urea and 1000 mOsm/kg osmotic salt ions. It can also maintain great stability at high temperatures in liquid, lyophilized, and immobilized forms for a long time. Katherine et al. exposed recombinant VNARs to mouse gastric scraping (pH = 5) and intestinal samples (proteinase-rich environment) and showed no evident signs of degradation after 1 h of incubation, thus demonstrating the stability of VNARs in extreme pH environments and their ability to resist proteinase hydrolytic cleavage [2,36]. In addition, Dooley et al. incubated VNARs at high temperatures (85–97 °C), and they still maintained binding activity after 3 h [31]. These characteristics provide great conditions for the development and improvement of mAb drugs and the potential of field immunodetection reagents [29,37,38]. Another advantage of sdAbs is the multiple ways in which they can be administrated, given their small size and stable biochemistry. For example, they can be directly entered into the lungs by aerosolization, which is particularly useful in combating respiratory viruses, such as SARS-CoV-2 [39]. 

#### 2.2.3. IgNAR Can Be Humanized

**Figure 3:**
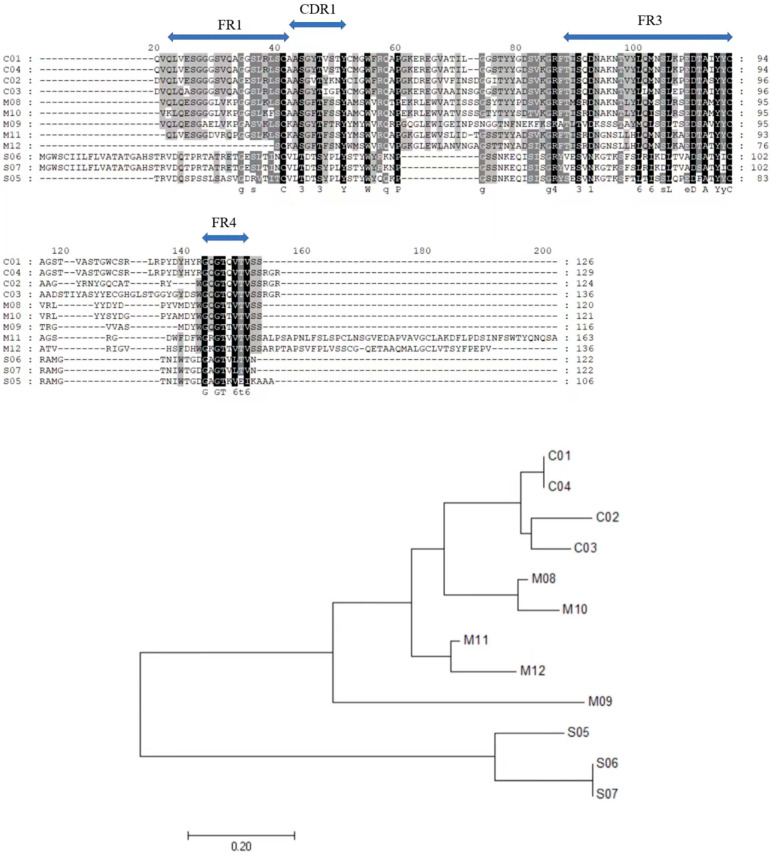
Sequence comparisons of heavy chains of antibodies from different species are shown, including the analysis of phylogenetic tree. C01–C04 stand for Camelidae VHHs, S05–S07 for shark VNARs, and M08–M12 for mice and other mammals’ IgG heavy-chain antibodies.

VNARs are distinct from typical Ig VH and VL domains as well as camelid VHH domains. They share higher structural homology with immunoglobulin VL and T-cell receptor V domains than with immunoglobulin VH [30]. The total area of 25–30% amino acid homology was found between VNARs and the variable region of human antibodies via sequence homolog analysis; the results showed low overall sequence identity [40] (Figure 3). At the same time, shark VNARs were conserved in certain key antigen-specific binding functional domains, thus suggesting that shark VNARs have antigen-binding properties that are similar to mammalian IgGs and VHHs. In addition, shark VNARs have their own unique sequence and structure, which may be different from other types of antibodies. However, based on the existing VNAR crystal data, the structure of VNARs has a high similarity to that of the variable region of human antibodies. Regarding in vivo therapeutic applications, shark VNARs need to be humanized to limit potential immunogenicity [41], as well as to improve thermodynamic stability, folding, and expression properties without reducing their favorable antigen-binding and structural stability characteristics [30,42]. CDR grafting is the most straight-forward and widely used humanization approach: the CDRs of a non-human antibody of interest are grafted onto an appropriate human germline framework, and the binding and functional properties of the original antibody are thus retained. 

In 2013, on the basis of screening specific VNARs [32], Kovalenko et al. focused on the VNAR domain of anti-human serum albumin (HSA), which was isolated from immunized sharks and could be humanized by converting over 60% of non-complementarity-determining region residues to a human germline sequence. In addition, within this process, the resulting molecules largely retain their specificity and affinity for the antigen binding of the parental VNAR [30]. In this study, conducting random mutagenesis on the resulting molecules was followed by the refinement of clones through an off-rate ranking-based selection of target antigens. After analysis, VNAR—which was selected in the aforementioned method—exhibited negligible antigenicity, high stability, and high affinity, which may meet clinical requirements [42]. Zhang et al. showed VNAR CDRs based on an analysis of currently available VNAR antigen structure complexes in the global Protein Data Bank archive of 3D structure data. They described a detailed protocol through which to humanize VNARs via CDR grafting [43]. Fischer et al. concluded that a structural and dynamic understanding of the VNAR binding site upon humanization is a key aspect of antibody humanization [44]. Based on existing research, the humanized transformation of VNAR can be realized via frame transplantation without losing functions. Meanwhile, Martin et al. proposed that, in this area, further work for VNAR humanization is needed to maximize human sequence content while avoiding a loss of binding affinity and/or immunogenicity that would result from aggregation or decreased stability [45].

### 2.3. Preparation and Mass Production of Specific VNARs

#### 2.3.1. Use of Chiloscyllium plagiosum as a Model Organism for VNAR Preparation

Early studies on sdAbs mainly focused on camelids because raising camelids is easier than raising sharks [46,47]. Contrary to mammals, the immune cycle in sharks is usually long [48]. Fewer than 10 shark species are currently being studied for the development of VNAR-immunized libraries, such as the nurse shark (*Ginglymostoma cirratum*) [7], wobbegong shark (*Orectolobus ornatus*) [49], and horn shark (*Heterodontus francisci*) [50], etc., with nurse sharks being the most common.

However, the generation of VNARs is hindered by the high cost and cumbersome care process of large shark breeding. They are difficult to maintain in captivity because of their endangered state, large body size, slow maturity, aggressive temper, and fast movement. In addition, certain cartilaginous fish fail to produce antigen-specific IgNARs, such as the small spotted catshark [51]. Compared with these options, *Chiloscyllium plagiosum* (white-spotted bamboo shark) is an attractive alternative ideal model to study and obtain antigen-specific VNARs through immunization as it has stable VNAR production, easy breeding conditions, as well as better maneuverability and repeatability of antigen immunization. 

Bamboo shark is a small, demersal species that seldom bites, has a strong body shape, and matures quickly [52]. It can be fed with an artificial diet, and it is easy to keep [35] and immunize in a laboratory environment [53]. Found in their genome, transcriptome, and plasma, bamboo sharks are the first shark species whose chromosomes are the complete configuration of all IgNAR family structures [35]. The recent discovery of shear forms in bamboo sharks has broadened the understanding of IgNAR properties [29], and their genome has revealed chromosomal rearrangements and the presence of rapidly evolving immune genes in cartilaginous fishes [54]. The presence of strong and rapid IgNAR recall was also observed when undergoing a response following a re-encounter with antigens. Many biological explorations on bamboo sharks, which serve as a promising small animal model for high-affinity sdAb generation, have been conducted [26,35,55,56,57].

#### 2.3.2. Phage Library Screening of Specific VNARs

The production of sdAbs is important for the detection and identification of potential candidate proteins for further drug studies [15,39]. In recent years, it has been demonstrated that sdAbs are highly amendable to a variety of modifications without losing their structural integrity; in addition, they are compatible with a variety of high-throughput screening platforms such as yeast [58], phage [27], bacteria [59], and ribosome displays [60,61]—with the phage display being the most established one because of its large storage capacity, simple operation, and the ability to be manipulated at the gene level. Meanwhile, besides the employment of phage libraries derived from immunized specimens, naïve [15,62] and synthetic libraries [63,64,65] have been explored as additional, less time-consuming options. It needs to be emphasized that IgNARs have experienced iterative affinity maturation in immunized sharks upon immunization [18]; therefore, VNARs from immunized libraries generally have higher affinities and specificities than those from semi-synthetic libraries and naïve libraries.

**Figure 4:**
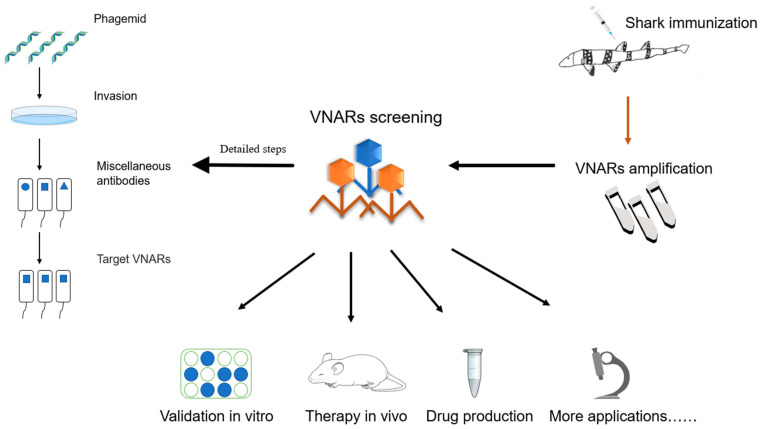
VNAR production using a phage display library. Specific antigens were used to immunize sharks and induce an immune response for approximately three to six months. After which, the screened VNAR sequences were cloned into phage vectors for phage antibody display, and phage libraries were made. The antibodies identified after screening can be used to assess their potency in vitro and in vivo.

Currently, the most popular and broadest approach through which to isolate target antigens for shark VNAR is through phage display (Figure 4). Using this technology, the gene encoding the antibody binding domain is fused with the phage coat protein gene to display the antibody on the phage surface for the selection of antigen recognition binding proteins [66]. Existing phages are built from stock in two forms. First, after repeated immune processes, simple blood samples are collected from animals to enhance and extract RNA, and then the VNAR sequence is amplified from the cDNA generated from the total information [67]. Antibodies with strong specificity and high affinity for immune antigens can usually be obtained with this method. Second, natural or semi-synthetic phage display libraries are constructed based on the VNAR domain produced by natural animals, and these usually contain additional diversity in the CDR region [68,69,70,71]. Feng et al. [15], based on EASeL technology, extracted RNA from six nurse sharks to construct a phage display natural library with a library capacity of up to 1.2 × 10^10^, from which antibodies with high affinity and those with a specific recognition of tumor treatment-related antigens and viruses could be obtained. 

#### 2.3.3. Recombinant VNAR Production

Duan et al. [72] transformed phages containing antibody sequences into expressing the HV2151 strain instead. In addition, they obtained large amounts of VNAR from the bacterial liquid lysate via purification in order to compare how to obtain sdAbs with high yield, quality, and purity. At the same time, they attempted to produce sdAbs using the mammalian cell line Expi293F. The advantage of mammalian cell expression is that it allows posttranslational modifications, especially glycosylation. However, monodomain antibodies do not require post-translational modification, and recombinant VNAR is highly soluble [73] and stable because hydrophilic residues are present on its surface. It also lacks glycosylation modification, which is easy to clone and express in bacteria [38], and it does not cost much compared with the production of traditional monoclonal antibodies [74]. Experiments have proven that the small-scale and large-scale production of recombinant monodomain antibodies have excellent biological functions [72]. Thus, the bacterial expression system is suitable for the production of large quantities of recombinant monodomain antibodies [75]. Experiments have proven that the yield of shark VNARs in Escherichia coli ranges from 2 mg/L to 15 mg/L [72] and that such a low cost can bring considerable economic benefits.

## 3. Potential Applications of VNAR in Preclinical Test

Intact mAbs dominate clinically approved therapeutic drugs. However, due to the inherent characteristics that limit the development of these antibodies, antibody fragments that are smaller and easier to permeate tissues have been developed for in vivo imaging and clinical treatments [2]. In terms of drug targeting, based on their ability to interact with demanding epitopes such as receptor domains, sdAbs have gradually become promising candidates for biomedical applications [76], many of which have been proven successful in early-stage clinical trials [60,77,78,79]. The drug delivery system, given the unique properties of sdAbs (Table 2), can maximize the delivery of drugs to the necessary sites and minimize off-target toxicity. VNARs have been modified and adapted to satisfy the requirements of drug discovery and development. Through this, a number of important additional attributes have been demonstrated, thereby leading to the belief that it will also become a valuable platform for multiple medical treatments [2].

### 3.1. Advantages in Drug Development and Clinical Treatment

When combined with traditional antibodies, VNARs have high affinity and clearly high tissue penetration. Based on its unique structure, the potential of VNAR technology as a powerful “engine” for novel drug discovery is evident. The small size and unique topology of shark VNARs provide an opportunity through which to penetrate tissues and bind to novel and hidden targets that are difficult to access through the larger spherical binding domains that are currently available. VNARs demonstrate a surprising ability for specific and high-affinity binding to diverse antigens by using just two variable domains per molecule. VNARs have evident advantages in developing highly specific and effective drugs against specific targets given their ability and versatility [80]; they also provide a new idea for the development of bis-specificity antibodies because VNARs can be easily fused [81].

Rapid distribution and elimination are desirable features of disease-specific biologics [82]; as such, the small size of VNARs provides extensive applicability for in vivo diagnostic imaging. Therefore, their excellent molecular characteristics make VNAR a potential substitute for the defects of traditional antibody drugs, thus allowing them to become a highly effective new targeted therapy agent. The screening of semi-synthetic [83] and CDR3-randomized VNAR libraries allows for the rapid and convenient identification of anti-idiotypic VNAR domains against monoclonal antibodies. The resulting VNAR variant does not cross-react with unrelated antibodies and was able to maintain its excellent target recognition in human and mouse sera. Molecular engineering and phage display technologies could be potentially used in molecular imaging, drug delivery, and the treatment of several major diseases [84].

Due to the large number of recombinant VNAR proteins that can be expressed in prokaryotes and the low cost of preparation, although there are still many challenges for the development of VNAR-based drugs, such as the need for humanization of the sequence and the short half-life of the vector, the price is much lower than that of traditional antibody drugs, and this serves as the basis for their widespread use. Increasing numbers of next-generation antibodies and novel scaffolds have been introduced into the research of biotechnology and biopharmaceutical companies to overcome the limitations of traditional antibodies, as well as provide additional opportunities for new disease-targeting and alternative drug delivery methods. 

The advantages of VNARs, such as their stable quality, low production cost, easy storage, and long half-life, are beneficial to the efficacy of their clinical applications. Ongoing preclinical developments, such as immunoassay [85], medical imaging [86], in vitro diagnosis [87], and disease treatment [88], will help define the utility of shark VNARs as a novel family of drug candidates for treating cancer and other human diseases [14].

### 3.2. Exploration and Development of VNAR-Based New Drugs 

VNARs may be an effective neutralizing agent against mutated virus strains based on their ability to bind highly variable influenza viruses (which cause great immunogenicity). In 2021, Gauhar et al. [89] reported the identification of neutralizing single-domain VNAR antibodies that were selected against the severe acute respiratory syndrome coronavirus 2 spike protein (which was derived from the Wuhan variant with the phage display approach). And then, Obinna et al. [27] and Valdocino et al. [90] compared the binding of VNARs that were selected from phage libraries and mutant virus strains. It was shown that the mutation of the virus had been proven to barely affect the affinity of VNARs. A crystallographic analysis of VNARs found that they recognized separate epitopes on the RBD and had distinctly different mechanisms of virus neutralization—ones that were unique to VNARs. VNARs were reported as showing picomolar affinity binding and a wide range of neutralizing activity toward the receptor binding domain (RBD) of SARS-CoV-2 variants [91]. In addition, they can be isolated from the SARS-CoV-2 RBD-immunized *Chiloscyllium plagiosum* [26] due to their ability to cope with evolving and evading COVID-19 [7]. The intranasal administration of VNARs can effectively protect mice from the challenges of SARS-CoV-2, as demonstrated by their advantages of small size and high bioavailability [91]. These results showed that VNARs offer unique binding capabilities to the RBD protein, especially in regions that are not readily susceptible to conventional mAbs. Thus, they should be a useful adjunct to existing antibody approaches in terms of treating COVID-19 as detectors and in therapeutics. Broadly speaking, these findings also suggest the potential druggability and assay reagent iteration of VNARs, which would provide these molecules with the potential for extensive use in human diseases.

Currently, three companies in the world are engaged in the research and development of shark antibody drugs: Ossianix, Elasmogen, and AdAlta. Only Ad-214 from AdAlta has been used in clinical practice thus far [92]. Ad-214 is an i-body developed by AdAlta that specifically binds to CXR4. Meanwhile, Ad-314 is fused with human Fc for the treatment of idiopathic pulmonary fibrosis. A phase I intravenous administration safety study has been successfully completed. At the same time, AdAlta is working on developing Ad-214 inhalable preparations so as to improve the bioavailability of the drug, improve patient convenience, and reduce the cost. Non-immunoglobulin VNARs were fused with anti-hTNF-α biologics (Quad-X™ and D1-NDure™-C4) to determine the effect of anti-drug antibodies (ADAs) on preclinical in vivo efficacy [75], and the promising applications of VNARs in the biomedical industry were also indicated.

### 3.3. Application of VNAR as an Enzyme Inhibitor

When antibodies are used as enzyme inhibitors, they can inhibit enzyme activity by changing the conformation of the active site(s) or directly binding to the active site(s) of the enzyme [93]. Burgess et al. [94] identified a VNAR that specifically recognized Aurora-A kinase by constructing a VNAR semi-synthetic library, and proved, via crystal structure analysis, that the VNAR could destroy the salt bridge, change the conformation, and inactivate the enzyme. The goal of replicating the precise targeting specificity of the antibodies and increasing added value through differential qualities, such as small size, has been the core aim in the development and progress of several alternative drugs in recent years. 

### 3.4. VNARs Can Penetrate Tissues and Deliver Drugs across the Blood–Brain Barrier

A well-defined high tissue penetration was verified in VNAR treatment. Drug delivery across the BBB remains an important barrier for the development of biopharmaceuticals with therapeutic effects on the central nervous system. On the basis of the small size of VNARs and their extended CDR3 loop, Pawel et al. [95] screened the synthetic VNAR phage display library for antibodies that can penetrate the brain parenchyma of mice and can be specifically taken into TfR1-positive neurons to address issues such as retention when using TfR1 receptor antibodies to cross the BBB. In subsequent assays [96], they demonstrated that the TfR1-targeting VNAR shuttle protein could be fused with the agonist antibodies, as well as be effectively transported to the BBB and delivered into the brain parenchyma at physiologically relevant concentrations. In addition, systemic treatments with VNARs can prevent neuronal loss. The experimental results proved that VNARs can rapidly cross the BBB with excellent pharmacokinetics and safety and that they can adapt to carry various biological therapeutic drugs from blood to brain at any time, thus making them the first of the next generation of brain-penetrating agonist antibodies with therapeutic potential in a wide range of brain diseases. 

Given the special structure of the corneas, Kocaleva et al. [97] administered the drug to the mouse model of corneal scratches via dropping; they proved that VNARs have the ability to penetrate the cornea and overcome the drawbacks of traditional antibodies, such as the fact that they could only be administered via vitreous injection, accompanied by infection and retinal detachment. Camacho et al. [98] also proved that VNARs penetraurface abrasion or discomfort and that they may become a new drug candidate for the treatment of vascular ophthalmopathy. These findings suggested that VNARs have good adaptability and penetration to host tissues.

The success of these applications showed that VNARs are druggable after perfect design and adjustment and that they satisfy the requirements of drug discovery and development, thus ensuring that VNARs could become a valuable platform for various medical treatments.

## 4. Applications in Anti-Tumor Preclinical Studies

Antibody drugs are mostly used in the treatment of cancers, autoimmune diseases, as well as infectious diseases, cardiovascular diseases, and organ transplant rejection. Antibody drugs are widely used in clinical treatment as targeted drugs; in addition, they account for a large proportion of the new FDA-approved drugs. Cancers and chronic inflammatory diseases are currently the main indications for antibody therapy, and this is in part due to the systemic accessibility of the target antigens [2]. As an emerging tumor treatment in recent years, immune antibody therapy can improve the ability of the immune system to recognize antigens on the tumor surface by changing the expression of immune regulatory factors in the tumor microenvironment [99,100].

**Figure 5:**
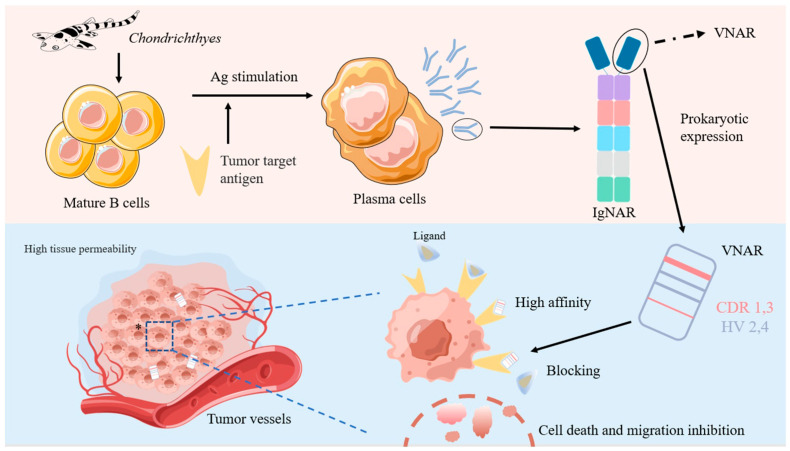
The production of IgNAR and its advantages and applications in the treatment of solid tumors.

The main limitation to the wide clinical use of ADCs is their non-uniform distribution in solid tumors. Another major factor affecting the efficacy of ADCs is the heterogeneity of their intratumoral distribution [101]. In addition to the complicated and harsh tumor microenvironment [102], massive neovascularization [103], variable antigen expression [104], and low clearance rate [105], the physicochemical characteristics of monoclonal antibodies—such as their large size and high binding affinity—slow down the penetration of these drugs into tumors [106,107]. Improving the intratumoral distribution of ADCs is critical to increasing their in vivo efficacy [108]. Early diagnosis is particularly important for the timely detection and treatment of diseases, especially for serious diseases such as tumors. The utilization of VNARs against various tumor markers has also been successfully developed to date (Figure 5).

### 4.1. VNARs Have Stronger Affinity to Cancer-Specific Targeting Antigens

Torchia et al. [83] chemically synthesized a small peptide through phage display library screening and then attached it to the amino terminus of a pre-prepared IgG Fc protein. They identified a small peptide with an affinity for a unique tumor type and demonstrated its ability to kill tumor cells specifically and trigger macrophages to phagocytose tumor cells. The effective clearance of human lymphoma in a mouse xenograft model demonstrated that this approach can be used to personalize and precisely target tumors. On this basis, Arturo et al. [65] screened VNAR libraries that were displayed by yeast through fluorescence-activated cell sorting (FACS) so as to enrich antigen-bound VNARs against the BCRS of different lymphoma cell lines. Five VNARs were expressed as Fc fusion proteins and revealed binding constants in the low one-digit nanomolar range.

Meanwhile, a proof-of-concept study based on the generation of VNAR antibody–drug conjugates has been reported. The high specificity of VNAR antibodies provides a therapeutic window for the eradication of lymphoma B cells, and future experiments may result in VNAR-Fc antibody formats with a higher binding valence. Treatment with VNARs has many advantages over monoclonal antibodies [60]. VNARs have a much smaller molecular weight than monoclonal antibodies, and this may allow greater tissue permeability. A molecule in the form of a polypeptide body can carry more than two antigen-binding domains, thus resulting in a higher affinity for the target.

### 4.2. Strong and Rapid Tissue Permeability

As potential chemotherapeutic agents, sdAds may passively target and penetrate tumor tissue with their excellent permeability, all the while their clearance by the lymphatic system is reduced. Adam et al. [109] proposed a new concept in the nanoparticle targeting of sdAds, i.e., anti-DLL4 VNARs were specifically conjugated with a target in DLL4 and used as a new treatment for pancreatic cancer. The studies demonstrated that VNARs can specifically bind to DLL4 with high affinity and are preferentially internalized by pancreatic cancer cell lines and endothelial cells that express DLL4. In addition, anti-DLL4 VNARs have significant anti-angiogenic effects. Arturo et al. [65] screened a yeast library for soluble anti-idiotypic VNAR targeting lymphoma cells and generated VNAR antibody–drug conjugates that induced lymphoma cell-specific killing at low concentrations of ~20 pM to 1 μM without significant cytotoxicity—even at higher three-digit nanomolar concentrations.

### 4.3. The Next-Generation Antibody for Anti-Tumor Applications

In 2017, Ubah et al. [110] immunized nurse sharks and screened anti-rhTNF-α (a recombinant homo tumor necrosis factor) sdAbs via phage display technology. An in vitro assay confirmed that the candidate VNAR obtained was as effective as adalimumab in the treatment of intestinal epithelial barrier dysfunction. In the follow-up animal experiments in 2019, the mouse arthritis model (Tg197) even indicated that VNARs are more superior to IgG in their in vivo binding capacity [111]. In 2022, Zhao et al. [53] identified 15 specific mouse TNF VNARs from a phage display VNAR library after immunizing a bamboo shark. Their findings indicated that VNARs showing affinity to mTNF-α were successfully enriched and identified by iterative biological screening. These results showed that VNARs can fulfill the requirements for next-generation antibody drugs in clinical tumor treatment.

Favorable therapeutic effects have been achieved in hematological malignancies since the introduction of chimeric antigen receptor (CAR) T cell therapy in this field. However, solid tumors remain a challenge due to the lack of appropriate antigen targets and immunosuppressive tumor microenvironments.

VNARs can overcome the disadvantages of traditional IgG drugs and effectively penetrate solid tumors with the help of their small size and high antigen-binding affinity. The PD-L1 immune pathway is crucial for tumor cells to escape from immune surveillance in the human body. Broos [112] stated, in their publication in 2019, that the antigen-binding component of this pathway should be further developed. Hpd-l1-binding sdAbs were prepared and injected intravenously in mice, and results confirmed that this antibody was able to block PD-1/PD-L1, enhance the T cell receptor signaling pathway, and kill tumor cells. Subsequently, bivalent and trivalent sdAbs were constructed using a glycine-serine linker, and their activities were 313 and 135 times those of a monovalent single-domain antibody, respectively. In addition, an attempt was made to link the identified high-affinity sdAbs fragment to the human IgG Fc fragment, and this modified Fc domain was found to effectively enhance the anti-tumor effect [113].

Dan Li et al. [20] constructed a semi-synthetic VNAR phage library and identified anti-PD-L1 sdAbs. These antibodies showed cross-reactivity with human, mouse, and dog PD-L1s, as well as partially blocking the interaction between human PD-1 and PD-L1. Through this process, to a certain extent, the prevention of tumor metastasis and the induction of tumor regression were achieved.

## 5. Conclusions

The progress of research into sdAbs as valuable drug conjugates is summarized in this review. The structural characteristics and potential applications in clinical tests, as well as the anti-tumor drugs, were highlighted to reveal the value of sdAbs as biomedical reagents. Biological agents predominate the market for therapeutic drugs, and innovation drives the development of the next generation of medical products. The development of therapeutic antibodies has focused on smaller, more stable, and more flexible products to improve the efficacy of the drugs and expand their clinical use. The structure of shark sdAbs brings about specific functions for these molecules, including a small size, excellent thermal stability, penetration into tumor epitopes, and a low production cost. These advantages will hopefully lead to the development of highly specific, stable, and effective drugs in the future [114]. These characteristics will enable sdAbs to become the focus of various clinical uses.

Shark-derived sdAbs and their conjugates can still be improved in terms of their stability, expression level, protease resistance, and the aggregation caused by synthetic linkers. Small-size proteins are usually associated with short serum half-lives. Generally, any molecules smaller than the threshold for glomerular filtration (approximately 60 kDa) will be rapidly eliminated from the body. Fusion to the Fc domain can solve this problem. However, if a small size is advantageous, then the addition of several extra domains (i.e., Fc) would counteract the advantage of a small size. Although a large number of preclinical studies on shark-derived sdAbs have been conducted, their progress into clinical use has been slow. Considering the great evolutionary distance between cartilaginous fishes and humans, potential immunogenicity should be considered prior to clinical application. However, the feasibility of humanizing VNAR domains to prevent xenoreactivity has been demonstrated in previous studies [2]. Although Fc fragments lack the ability to mediate the ADCC effect and to guide immune cells to clean the targeted tumor cells, VNAR structural characteristics and their high affinity for target antigens make it possible for them to be used as effective antigen-binding therapeutic drugs.

This study also indicated that bamboo shark, as a model organism for the preparation of high-affinity VNARs, may contribute to paving the path for VNAR application as a clinical drug candidate. However, in the process of IgNAR preparation, identifying methods to dynamically monitor IgNARs in sharks for observation and recording is still necessary.

The main direction of antibody development at present is to reduce the size and complexity of antibodies while maintaining their affinity and selectivity for their targets. This approach includes natural antibodies (such as single-domain antibodies), antibody-like domains, and scaffolds. sdAbs should still be modified and clinically verified for their aspects, such as with respect to their immunogenicity and functionalization, even though these antibodies can efficiently expand potential drug targets due to their unique structural characteristics [84]. Envafolimab is the first camel-derived humanized PD-L1-IgG Fc single-domain fusion antibody drug to enter phase I clinical studies [9]. However, drugs based on shark-derived VNARs have rarely been clinically studied. The mechanism of action of VNARs with their molecular targets in vivo should be further investigated for VNAR antibody drugs to clarify their function, benefits, and efficacy in tumor immunodiagnosis and treatment. Shark VNARs are promising in the treatment and early diagnosis of tumors, especially solid tumors, due to their superior molecular structure characteristics and their objective advantages over VHH. Further development of VNAR antibody drugs may change the future of biological agents.

## Figures and Tables

**Table 1:** Comparison of the advantages and disadvantages of the V region of mAbs, VHHs, and VNARs.

		V Region of mAbs	VHH	VNAR
Structural complexity	Complex post-translational modifications (glycoproteins)	+++	—	—
	Expression in prokaryotic cells	—	+++	+++
Molecular weight	molecular weight (~kDa)	50~75	~15	~12
	Tissue penetration (BBB, ocular model, placental barrier)	—	++	+++
Structural stability	Physiological and biochemical environment (high temperature, pH, urea)	—	+++	+++
Shortcomings	Difficulty of humanization	—	+	+++

In the comparison of strengths and weaknesses, +++ represents the highest degree, + represents a slightly weaker ability, — represents no or less ability.

**Table 2:** The first marketed single-domain VHH-based antibody drugs and current VNAR-based drugs in development.

Drug	Application	Source	Clinical Trial Phase	Ref.
Caplacizumab	aTTP	VHH	2019 FDA-approved	[76]
AD214	Idiopathic pulmonary fibrosis	VNAR	Phase I	[77]
TXB4	Primary central nervous system Lymphoma	VNAR	Preclinical	[78]

## Data Availability

Not applicable.
